# Experiences of Blogging About Visible and Long-term Skin Conditions: Interpretative Phenomenological Analysis

**DOI:** 10.2196/29980

**Published:** 2022-04-22

**Authors:** Selina K Tour, Andrew Thompson, Ruth A Howard, Michael Larkin

**Affiliations:** 1 School of Psychology University of Birmingham Birmingham United Kingdom; 2 South Wales National Health Service Clinical Psychology Training Programme Cardiff University Cardiff United Kingdom; 3 School of Life and Health Sciences Aston University Birmingham United Kingdom

**Keywords:** peer support, blogging, psychodermatology, stigmatisation, emotional disclosure, self-management, qualitative research, interpretative phenomenological analysis

## Abstract

**Background:**

Skin conditions can detract from people’s quality of life, much like conditions such as cancer, chronic pain, and depression. Visible skin conditions can lead to risk of stigmatization. It is acknowledged that there is a lack of available psychosocial support for people living with chronic skin conditions. One way in which individuals with long-term conditions are self-managing and providing peer support is through blogging and exchanging information on the web. To date, no research has specifically investigated how individuals with skin conditions experience the use blogging for self-management.

**Objective:**

This study sought to investigate the experiences of individuals with visible, long-term skin conditions when blogging about their conditions.

**Methods:**

A systematic blog search and a short survey were used for recruitment. A total of 4 participants took part in email interviews, which were analyzed using interpretative phenomenological analysis (IPA). Skin conditions included alopecia, psoriasis, and hirsutism. The content of these individuals’ blogs was also analyzed using a qualitative template method derived from the IPA analysis.

**Results:**

The interviews and accounts revealed a clear sense of uncertainty about the course of the bloggers’ skin conditions. This appeared to be associated with feelings of distress and isolation, searching for treatments, and ultimately a sense of defeat. The data revealed that blogging provided a space where this sense of defeat was managed and challenged. Posting on the web facilitated connection with others and enabled support networks to be established that assisted in challenging the feelings of isolation experienced. The data demonstrate the important role that blogging played for these participants in developing a sense of acceptance of their condition.

**Conclusions:**

Blogging may provide a way for individuals to self-manage distress associated with visible skin conditions. It may provide similar benefits to those known to be derived from emotional disclosure that occurs during writing, with an added peer support dimension. Blogging has occurred naturalistically on web-based forums, and this study demonstrates how this form of interaction may warrant adaptation for use with web-based psychosocial interventions for people living with skin conditions. This study had a limited sample of 4 bloggers; therefore, further exploration would be needed to consider the utility of this approach.

## Introduction

Skin conditions, such as atopic dermatitis, psoriasis, vitiligo, and urticaria, can be characterized by their long-standing, incurable, and dynamic nature [1]. Such conditions require continuing care [2] and can have an impact on quality of life similar to that of other long-term conditions (LTCs) such as heart disease, cancer, diabetes, and depression [3-6]. Skin conditions are known to be associated with levels of depression, anxiety, and suicidal ideation that are higher than those in the general population [7,8].

The visibility of skin conditions can be associated with additional distress [9,10]. Since individuals not only have to manage the symptoms but also the reaction of others [9,11], it is not surprising that these individuals can experience psychological distress. However, there is an acknowledged lack of available support for the ongoing emotional and psychosocial distress associated with having a skin condition [12,13].

The internet provides further opportunities for individuals to express themselves. For example, people have been found to write about personal experiences, ask questions, and receive direct feedback when discussing diabetes on moderated Facebook forums [14]. Different digital spaces offer different opportunities for emotional disclosure. For example, forums are typically moderated, and thus some forms of expression are restricted. Blogs are web-based journals where individuals can write their thoughts in a chronological format, to connect with others in a peer-led environment [15,16]. Blogs are not externally moderated, so bloggers can share their experiences in whatever form they wish; as such, they provide “cleaner” access to how people living with skin conditions may wish to use the web-based environment.

Through blogs, individuals with chronic conditions have been found to experience decreased isolation, be more able to make sense of their condition, gain support, and feel a sense of belonging [16-19]. However, little is understood about the personal benefits of blogging: why people turn to blogging, how it impacts their sense making, or how they self-manage through blogging [18-20]. Our study aims to explore the personal experiences of blogging about a long-term skin condition which affects one’s appearance, and to consider this alongside analysis of the blog content.

## Methods

### Overview

In our study, we used two forms of data collection (direct email interviews and indirect blog content) and two forms of phenomenologically informed data analysis (interpretative phenomenological analysis [IPA] [21] and template analysis [TeA] [22]) to explore experiences of blogging about skin conditions.

Email interviews were chosen after consultation with two bloggers who blog about their visible skin conditions.

### Sampling

A combination of different research terms was used to find the blogs using the web-based search engine Google. This search identified 37 suitable blogs.

Blogs were required to be written in the English language, have been active for at least 6 months, have at least 10 entries, and have been posted on the web within the last 3 months. Exclusion criteria included carers or parents writing about skin conditions and blogs primarily advertising or writing about other topics, including cancer.

The 37 potential participants were invited to complete a short web-based survey via email. The survey provided study information and a consent page for potential participants to complete prior to questions assessing their suitability.

### Participants

Inclusion criteria for the study were having a skin-related LTC that was the primary motivation to start the blog and that was visible to others (ie, on the face, neck, or hands). Within this study, LTCs were identified in line with the World Health Organization’s definition: health conditions that persist across time and require some degree of management.

A total of 11 individuals completed the survey, 8 of whom met the inclusion criteria. A total of 4 bloggers participated in the research (a sample size appropriate for IPA [21]): 2 women and 2 men, aged between 24 and 45 years. A total of 4 individuals did not respond to the interview invitation. Participants were based in three different countries (the United Kingdom, Canada, and Australia), but all identified as White British/other. Their conditions had been present for between 2 and 10 or more years and included alopecia, psoriasis, and hirsutism. The bloggers had been blogging for 2 to 10 years. The bloggers all engaged with their blogs through writing posts, reading comments, and responding to readers.

### Procedure

Participants were invited to take part in a semistructured email interview with the first author. They received a £10 (US $13.50) Amazon voucher for their participation. The interviews involved an email exchange that was limited to 10 emails within a time frame of 6 weeks.

### Ethical Considerations

Although blog content is freely available on the web, participants were asked to give consent for their blog data to be used within the study.

Steps were taken to ensure the anonymity of the interviews. For example, the quotes included do not detail the participant’s condition or highlight distinguishable features of one individual, such as special events that may map onto a blog.

In addition, without verbal or facial cues, the ability to notice distress or need for support may be limited for email interviews. Participants were sent a list of support options (country-specific) that were available to them if needed. No participants reported any distress to the interviewer.

Our study was approved by the research ethics committee at the University of Birmingham (ERN_16-1472).

### Data Collection

#### Email Interviews

Participants were provided with the interview schedule prior to the beginning of the study and then asked the initial question with prompts to begin the interview exchange. Questions focused on the participants’ experiences prior to blogging, experiences of seeking help, and reasons for initiating the blog, as well as the role of blogging in living with their skin condition. The full interview schedule can be found in Multimedia Appendix 1, and it follows guidance for developing questions for an IPA study [21]. The interviewer would respond to the participant’s response with questions to further explore, gain clarity on information provided, and elicit further information. The email interview was asynchronous and guided by the pace of the participant.

#### Blog Content

The first five and most recent five blog posts of the bloggers interviewed were used for the purposes of TeA.

### Data Analysis

The first author conducted the IPA and TeA. Interview transcripts were read in turn and initial notes were made. A second read-through focused on line-by-line coding of objects of concern, tone or feeling, and language used [21]. A case summary was created for each participant to bring together notes, reflections, and codes. Codes across participants were then themed through their connections and associations by the first and last author.

The template for TeA was created using the preliminary IPA findings to align with the a priori defined themes approach of TeA. Subsequent revisions of the template were developed during the analysis of the blog transcripts. Codes across participants were linked and themed through their connections with one another and further refined.

The email interviews produced an average of 31 pages of double-spaced and wide-margined text for analysis (range 18-46 pages). The blog content analysis produced an average of 34 pages of text per blog (range 21-45 pages).

Research supervision with the second and last author helped to maintain the rigor of the research and coherence with IPA and TeA processes. The third author (external to the design and analysis) checked the quotes and themes for credibility. Participants were sent the preliminary themes and findings; one participant responded to the email to confirm that this fit with their understanding of their skin condition and blogging, as well as to ask for the final write-up. Other participants did not respond.

## Results

### Overview

The results presented here focus on the experience of blogging about skin conditions*.* We begin by briefly presenting the thematic structure from the TeA of the blog content. The template included the themes of symptoms, making sense of the condition, social experiences, treatment, and emotional impact. We then focus on the themes from the IPA of the interviews, and we discuss the similarities and differences between the two.

### Blog Content Analysis

**Figure 1:**
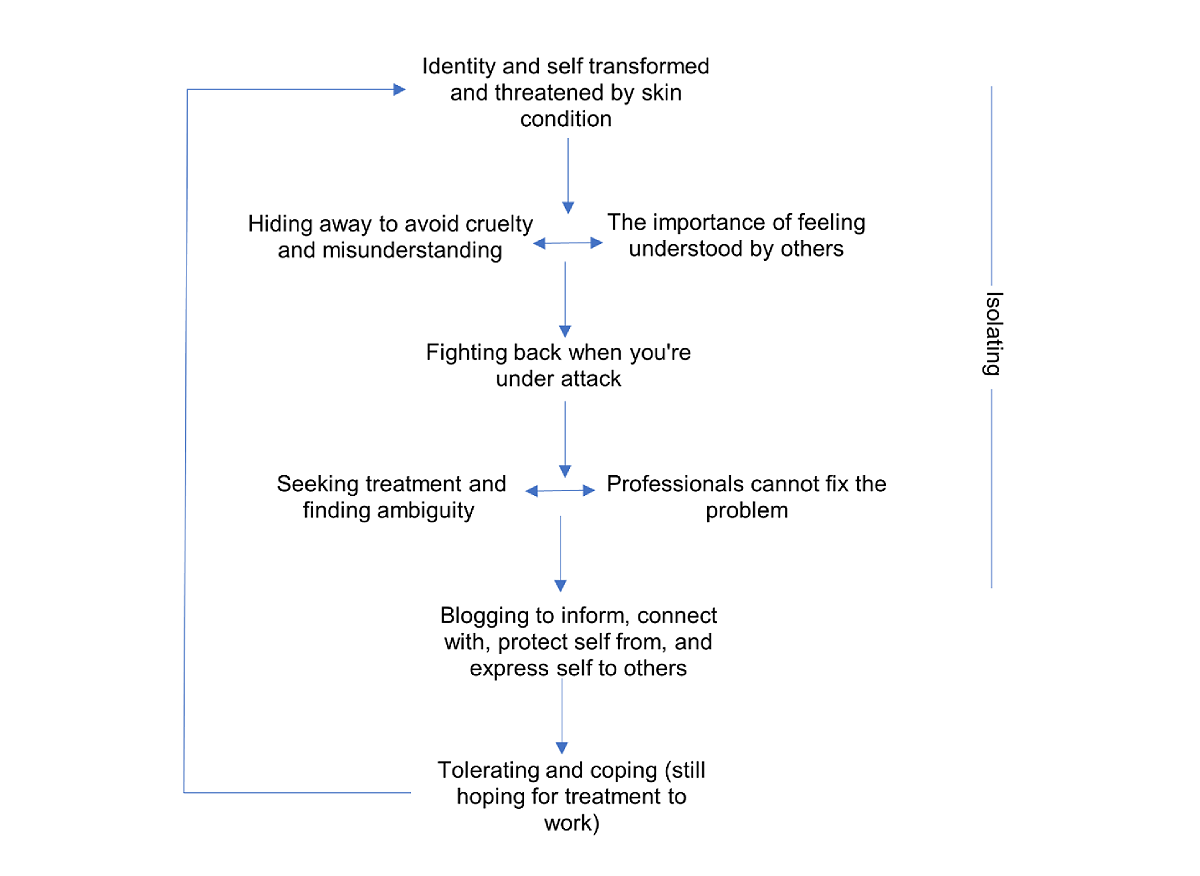
A visual depiction of the Template Analysis Theming structure.

Six main themes were drawn out from the TeA of the blog content. Figure 1 depicts the thematic structure captured by the analysis. The blog accounts of living with a skin condition did not follow a linear pattern; instead, participants fluctuated between different positions dependent on the state of their skin, relating to the cyclical nature of their skin condition. Participants attempted to manage the threat in different ways, such as hiding away and seeking treatment. This was intertwined with their experience of others. Blogging appeared to assist participants in moving from being overwhelmed by their condition to a place where it was no longer a threat. This did not reflect an acceptance but rather a tolerance of the condition, and it reoccurred each time the condition worsened. The participants therefore moved between “levels” of the structure dependent on the state of their skin and their social experiences with others. There was a general sense of this threat being an isolating journey, in contrast to blogging, which allowed connection and expression.

### Blogging as an Experience

Five superordinate themes were developed from the IPA. Table 1 outlines these themes along with subthemes, contributions, and quotes. Alongside the themes developed from the IPA, we provided some quotes from the blog content which map onto the themes. To maintain anonymity of the interviews within this paper, aliases are used for the participants, and blogs are labelled as 1, 2, 3, and 4. Further quotes can be found in Multimedia Appendix 1.

**Table 1:** Themes identified for blogging through interpretative phenomenological analysis and comparison with template analysis.

Superordinate theme and subthemes			Quotes from interviews		Quotes from template analysis
**Skin is an overwhelming threat to self**					
	Skin appraisal and attempts to manage	“I remember being self-conscious about my facial hair as an adolescent, when it was simply blond.... I vividly remember a boy commenting rudely on it in school, around the age of thirteen or fourteen, and that’s the first time I recall feeling like I should do something about it, even though I had been concerned about it for a while” [Laura]		“...two tiny little dots which I thought were just that – wee dots where hair didn’t grow. Until they started to spread. Obviously like any other person who cared about their looks I was worried – in fact I was majorly freaked out if truth be told. Bordering on daily obsession! As The ‘wee dots’ graduated into ‘big dots’ I tried to remain calm – while simultaneously obsessively checking the reaction of people I bumped into and whether they thought I had some human form of mange!” [Blog 1]	
	Defeat and discontent	“I felt that no one could help me and nothing could fix this” [Laura] “I was losing hope with the condition as no-one seemed to give me any answers” [Debbie]		—^a^	
**Blogging for self—venting and building**					
	Having an outlet and processing	“I knew it was interfering with things and causing some low moods and blue days. I wasn’t content … I couldn’t keep living life the way I was.” [Laura] “I find that when I talk about it – it is usually weighing on my mind because I am sad.” [Ian]		“I do feel that it has made me a stronger person and in many ways a very different person. I am by no means cured of my Alopecia but I am feeling more in control of it.” [Blog 3]	
	Being built up	“It was much more rewarding then what I initially thought. I did not expect the reception and the feeling I had from helping others was very rewarding.” [Debbie] “My management strategies must have changed. This is a little blurry but suffice to say that before blogging I don't think I had any. [It] was clearly winning and I wasn't managing it at all.” [Tom] “The more I wrote about the condition, and the more questions I got from readers, the more I noticed holes in my knowledge and it prompted me to do research.” [Laura]		“But in all my wanderings, nothing has helped so much as hearing from other women struggling with the same things” [Blog 2]	
**Blogging for others—sharing and informing**					
	Being there for others	“What was missing was the happy stories or the sad stories with a positive slant - or the fact that there could be people who coped fine with alopecia.” [Ian] “When I was writing to help others, I gained perspective and was distracted from my own problems, and they felt like much less of a burden” [Laura]		“As you may have realised after reading my first few blog posts, I decided to write this to help and support others and to bring attention to this crippling condition” [Blog 3] “I hope this gives the reader an understanding of what Psoriasis does to a person’s soul” [Blog 4]	
	Complement but not a substitute for the real world	“They do have a better understanding of why sometimes I want peace and quiet, why I am tired and they don’t have to ask how I am all the time. I guess my blogging saves them time and questions.” [Tom] “I may have missed the opportunity to take the risk of relying on others” [Laura]		—	
**Trying out a way to engage with others**					
	Safety in expression on the web	“It seemed less likely that I would be recognised amongst all that noise” [Laura] “I did not want to meet mass groups of other sufferers but I did still want help…. Blogging for me helps bring together communities of other sufferers without having to interact if you don’t want to.” [Debbie] “For me it is easier to tell a stranger and I think the reason is that I am not worried too much about how they think of me after as I'm unlikely to see them again” [Tom]		—	
	Social approval in blogging	“I am always wary not to be too negative as I don't think that is helpful. I am honest about the low times but don't want someone to be thoroughly depressed and leave my blog without some sort of hope.” [Ian] “I think I was very aware of how I came across and never wanted to appear too self-indulgent or negative.” [Laura]		—	
**Blogging as a journey which ebbs and flows**					
	When is the time to blog	“I see blogging as something I’ll continue to use as long as my condition persists.” [Ian] “I no longer had those powerful emotions I needed to let out. And I felt increasingly drained by the emails I received. I just felt ready for a life where I wasn’t forcing myself to think about [it] every day [...]. and blogging was the only thing keeping it ‘big.’” [Laura]		“It was something that I had expected and prayed wouldn’t come” [Blog 1]	
	Making the condition more tolerable	“Before I started the blog I saw psoriasis as this big dark monster looming over my life and in a way that view hasn’t really changed. Psoriasis has never been my friend and it never will be. Also unlike others who say they come to terms with it, I never have and refuse to do so. Mostly because I think if I do that then it has won. I perceive coming to terms with it as accepting it and I will never accept it, I want it gone.” [Tom]		—	

^a^—: no quote available.

### Skin Is an Overwhelming Threat to Self

#### Defeat and Discontent

Participants experienced a sense of defeat and a loss of hope when they realized that no treatment was working. There was an expectation of a cure, and participants described feelings of defeat when this no longer seemed an option. This was difficult for participants to face, although this was not reflected much in the blog content itself.

### Blogging for Self—Venting and Building

#### Having an Outlet and Processing

Participants described realizing that they needed to change the way they lived with their skin condition. Laura’s account of the turning point is typical: “I knew it was interfering with things and causing some low moods and blue days.  I wasn’t content … I couldn’t keep living life the way I was.” Blogs were described as a place where the participants could unburden themselves of these strong emotions. All participants described the *heaviness* of their condition and reflected on how their blogs were an outlet for these difficult emotions.

As they faced the difficulties arising from long-term illness, blogging provided opportunities for the participants to gain perspective and normalize. Participants described how these combined benefits helped them to find the strength to cope. This was particularly important for those who had conditions which fluctuated. As the condition worsened, the blog provided a way to chart the emotional changes. Participants described a growing sense of tolerance (rather than acceptance), as their relationship to the condition changed over time. In this way, the blogs served as a vehicle for managing the psychological distress and uncertainty associated with the skin condition.

#### Being Built Up

Through helping others, the participants described how their sense of accomplishment and competency grew. Laura described moving from a place of fearing the unknown to being more determined to find a form of control. Similarly, there was a sense of accomplishment from receiving positive feedback*.*

Participants also described the development of coping strategies through advice offered by readers. Thus, blogging was represented as a *scaffold* that helped participants to build themselves up emotionally after struggling to cope with the perceived sense of their skin condition being overwhelming.

### Blogging for Others—Sharing and Informing

#### Being There for Others

The participants expressed a desire to offer what was missing from their journeys to others. The participants attempted to normalize the condition for others. Being there for others meant that others did not experience the same frustrations they did. For Laura, blogging for others also provided a conscious escape from her own problems.

However, blogging was also seen as an exhausting process, because at times it meant supporting people who were often in a worse place. Laura described wondering whether her blog meant she never gained support herself. In this way, solely being there for others had the potential to prevent one from asking for or receiving help.

#### Complement but Not a Substitute for the Real World

It was clear that blogging did not replace a desire for interaction in the real world. Tom described blogging as an addition that helped his friends and family to better understand his experiences. Blogging supported Ian in connecting with others, whom he then met in person. It therefore helped him to grow his real-world network. In contrast, Laura described blogging as a barrier to connecting with people in the real world. Blogging did not appear to offer a viable *alternative*, but rather an *addition* to real-world contact.

### Trying Out a Way to Engage With Others

#### Safety in Expression on the Web

Participants described their anxiety around people knowing about their condition and receiving judgmental responses. Consequently, participants socially withdrew as their condition worsened. The fear of negative reaction appeared to be less threatening when writing on the web. Talking on the web provided an emotional distance when talking about a distressing condition. It felt safe to participants that they could choose when and how to respond.

Participants described feeling that no one understood—friends were distant, professionals were matter of fact, and family members did not always offer empathy. Blogging provided a way for participants to voice their frustrations and be understood without fear of retribution from a “real” person. In this way, blogging supported safe expression and emotional distance.

#### Social Approval in Blogging

This subtheme was noticeably missing from the blog content; however, during the interviews, it appeared to be integral to writing on the web. Although blogging provided an avenue where appearance was not important, there was often pressure to “say the right thing.”

Blogging was perceived to reduce appearance-related anxiety, and anxieties about saying the right thing were heightened. Sometimes, this caused more anxiety. Although blogging is not a face-to-face interaction, all participants described feeling the need to include a positive perspective; there was always a conscious awareness of those who might read the blog.

### Blogging as an Experience That Ebbs and Flows

#### When Is the Time to Blog?

As the participants’ skin conditions became more threatening, they used their blogs to manage the affect that came with it. Conversely, there appeared to be less need to blog when symptoms waned. Laura described how blogging eventually became a barrier. Interestingly, being there for others led her to maintain her web-based presence, although it no longer felt necessary for her personally.

#### Making the Condition More Tolerable

Blogging supported participants to feel less overwhelmed by the “looming” qualities of their conditions.

Participants described how blogging supported them in coping with overwhelming and unpredictable factors. However, it did not necessarily lead to feelings of acceptance. A hope for improvement remained; when the condition worsened, it was emotionally difficult. Participants used their blogs to make sense of the fluctuations but did not reach a place where worsening of their condition was easy to tolerate. However, participants also did not revert to their preblogging states: blogging appeared to offer a means of “containing” the affect that was once overwhelming.

## Discussion

### Principal Findings

Similar to experiences in other LTCs, blogging served as a way for participants to make sense of their emotions, adjust to their condition, and share information [16,23-26]. This appeared to compensate for the feelings of loss and isolation brought on by the condition and provided participants with a way to regulate their emotions [25,27,28]. Similar to findings from Johnston et al [29], this study suggests that distress may arise from relying solely on problem-focused strategies when facing an LTC. Participants used the blog at times when they needed support to move back toward a place of health [16,30,31], suggesting that blogging is a functional, but optional, tool in adjusting to LTCs for the participants.

Changes in mood and perspective appeared to be more prominent in the participants’ descriptions than changes in symptoms. Expressing emotional experiences through writing is associated with therapeutic benefits, such as positive health outcomes and reduced health appointments [32,33]. Thus, blogging about health conditions may be associated with similar benefits to emotional disclosure. This supports the idea of blogs as an emotion-focused strategy, as these strategies focus on changing the appraisal of a stressor that cannot itself be changed [28]. It is interesting to consider whether other web-based platforms (designed around image sharing or microexpressions) would be as well suited to supporting these reflective and connective strategies.

Negative impacts of blogging were also identified by the participants. The interviews identified a conscious awareness of audience and of readers’ response to their writing. Participants described the need for some positivity in their writing and not wanting to leave readers feeling worse. At times, they also felt a sense of obligation to blog for others even when not wanting to do so for themselves. It is not clear whether this detracts from the personal benefits of blogging or maintains them. The participants also experienced some sense of regret that blogging commitments may have prevented them having more face-to-face relationships. The social dimension of blogging therefore had both positive and negative impacts.

The use of email interviews is still relatively new to IPA research. Within this study, it fit with the participants’ sharing style (ie, writing on the web, anonymously), and we felt it was suitable for this population. However, this method can mean that verbal and nonverbal cues are missed, while it provides benefits such as fewer resources and more privacy [34]. Therefore, as with other methods, the advantages and disadvantages of this method need to be considered. One benefit of using interviews within this study was their ability to capture the personal benefits and challenges of blogging, which was not possible in previous studies focusing on content [16,35]. Within this study, the need for social approval in the context of writing on the web was highlighted in the interviews but not the blogs themselves. This suggests that what people post on the web and what they think about what they post are not identical in nature, and therefore research focusing on content alone may limit our understanding of the experience of writing on the web. Within this population, there is also a possibility that the need for social approval may relate to the visible aspect of conditions and the particular social difficulties this creates. However, this was not found within the TeA to be a shared experience with readers but more of a private experience. Further research would support a better understanding of the motivations for blogging for this group and how this differs from traditional emotional disclosure methods such as journaling.

When thinking about care practices within the United Kingdom, the stepped model of care proposed for psychodermatology services does not explicitly report a need for both emotion- and problem-focused strategies [13]. Problem-focused treatment is dominant at the initial stages of managing skin conditions. This study and other previous findings indicate the utility of more emotion-focused strategies for patients in addition to solely managing symptoms of an incurable condition. It may be useful to further explore support avenues for individuals to find emotion-focused ways of coping. This may include signposting individuals to existing forums and blogging sites or through creating bespoke peer support forums or public web-based journals that patients can use to both express themselves and engage socially with peers if they choose to. This may reduce the development of psychological distress [7] when conditions are incurable through drawing on literature from psychosocial interventions in other LTCs. However, it is worth considering that this study looked at the experience of using self-initiated blogs. The experience may be different if it is professionally led or organized. It would also be important to consider how people access such support and whether they would need to be receiving secondary dermatology services. The potential negative impact of writing on the web is also in need of consideration when thinking about its utility for individuals in their journey.

### Limitations

The sample of active bloggers within this area was relatively small, and although all their skin conditions were visible, the conditions were different. It will be helpful to explore self-management through web-based platforms for one condition and also on other platforms such as Instagram, where communities of individuals living with skin conditions use other methods such as photojournaling. Although photojournaling is a different type of blogging, it allows individuals to chronologically post, express themselves, and connect with others. A larger, more diverse sample may help us to understand whether emotional expression within a web-based community is a functional tool for adjustment across platforms.

### Conclusions

Blogging appears to share the benefits offered through emotional disclosure—with the added social dimension—and may support positive adjustment. Exploration of how blogging fits into current care and whether it would be a suitable self-help option to offer needs to be further considered. In particular, the social challenges of blogging may need further consideration, including the negative impacts of writing on the web. It still remains unclear how and under which conditions blogging can be successful in coping [36].

## Appendix

Multimedia Appendix 1 Interview schedule.

## Abbreviations

IPA: interpretative phenomenological analysis
LTC: long-term condition
TeA: template analysis
