# Supplementary material for: Experiences of Blogging About Visible and Long-term Skin Conditions: Interpretative Phenomenological Analysis
Source: JMIR Dermatol. 2022 Apr 22;5(2):e29980. doi: 10.2196/29980 (PMC10334883; doi:10.2196/29980)
Supplement: Multimedia Appendix 1 [file derma_v5i2e29980_app1.docx]

**Supporting Information**

**Semi-structured interview schedule**

**Can you tell me about your skin experiences before you began blogging**?

*Prompts: What can you remember about when you initially began to get symptoms? Did your skin symptoms change over time? How did it feel in your body? How did you interpret your skin condition? What was going on in your mind then?*

***Can you tell me about what happened when you began to seek help?***

*What kind of treatments did you think were appropriate? What did you think should be done to overcome your skin condition? What kind of support did you find helpful/ unhelpful?*

**Can you tell me about how you came to begin blogging?**

*Prompts: What was going on with your skin at that time? How were you managing at that time? How did you start blogging?*

***Can you tell me a bit more about your writing?***

*Prompts: When do you write? What do you write about? What have you learned? What has been challenging?*

**How would you describe the role played by blogging in living with your skin condition?**

*What is similar/ different about how you thought/perceived your skin condition prior to blogging? Have your management strategies changed or stayed the same? What would you say blogging is about? What role does blogging play for you? How has blogging impacted on your support networks?*
